# Reuse of nests in the Great Reed Warbler *Acrocephalus arundinaceus*: A behavior to save time and energy and to deter nest parasites?

**DOI:** 10.1002/ece3.9452

**Published:** 2022-10-27

**Authors:** Thomas Oliver Mérő, Antun Žuljević, Olga Kolykhanova, Szabolcs Lengyel

**Affiliations:** ^1^ Department of Tisza Research Centre for Ecological Research, Institute of Aquatic Ecology Debrecen Hungary; ^2^ Nature Protection and Study Society – NATURA Sombor Serbia

**Keywords:** brood parasitism, hatching failure, nest depredation, predator avoidance hypothesis, time/energy‐saving theory

## Abstract

The reproductive period in animals is a demanding part in their life history. In birds, environmental factors, such as adverse weather, predation, or brood parasitism; and/or anthropogenic disturbance, can limit breeding success, resulting in failure of clutches. The nest loss in open‐cup nesting passerines is usually replaced with a new nest with a new clutch, however, in some cases the clutch replacement may occur in unusual forms. In this study, we report on three cases of within‐season nest reuse in the Great Reed Warbler. In the first case, a nest was reused for two times in the same season after unsuccessful nesting attempts (two‐time nest reuse). After the nest was depredated the first time, the female laid new eggs that were depredated again, then again the female laid new eggs that produced four fledglings. In the second case, the first clutch was depredated, after which the female laid a new clutch in the same nest that was again depredated. In the third case, the female laid new eggs among the eggs that failed to hatch previously. Our observations tend not to be consistent with the predator avoidance hypothesis because the depredated nests were reused by the parents. The time/energy saving hypothesis or possible deterrence of nest parasitism could explain nest reuse in this study, but because of low number of nests reused compared to the total number of nests found, this phenomenon needs further clarification.

## Graphical abstract

In this study, we report on three extreme cases of within season nest reuse in an open nesting passerine. In first case, one nest successfully fledged young after the nest was reused for the second time (two‐time nest reuse), in the second case, one nest was depredated after nest reuse, and in the third case, the female laid a new clutch among the infertile eggs from the initial clutch. Our results were fairly supported by the predator avoidance hypothesis, they seem more likely to support time/energy saving and anti‐parasitic behaviour.

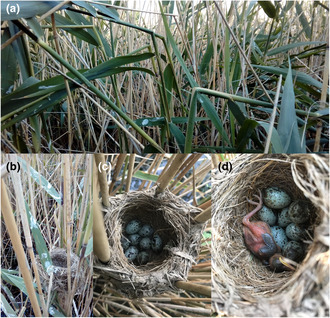

## 1 INTRODUCTION

The extent of parental care and investment varies across different animal taxa, while some species leave offspring on their own, others stay until young grow to be independent from their parents (Fromhage (2017). While some animals search for a shelter for offspring rearing (e.g., cavities, burrows) in their environment, others create shelters by constructing them (e.g., holes in the ground, nests; Hansell, 2005). Many insects, fish, reptiles, birds, and mammals construct nests with various designs in which they raise young (Hansell, 2000, 2005). An important determinant of breeding success is the selection of a safe nesting site, which may reduce the limiting factors, such as predation, food shortage, on brood rearing, and increase the fitness of offspring (Mainwaring et al., 2014). Although nests can provide safety, still a variety of environmental factors, such as habitat structure, weather conditions, predation, brood parasitism, resource availability, and parental behavior can influence the survival of clutches (e.g. Klug et al., 2009).

Rearing offspring in reused nests can be risky. In particular, nest predation can be risky for nest‐reusing pairs because predators often memorize earlier depredated nests and revisit them from times to times (Otterbeck et al., 2019; Sonerud & Fjeld, 1987). Reused nests can provide reduced quality of nest construction, being unable to hold the clutch (Mazgajski, 2007). Nests may become unstable during the first clutch, and may fall during reuse, mostly in adverse weather (Shields & Crook, 1987). During the first nesting cycle, ectoparasites and pathogens may accumulate in nests, that can cause mortality to clutches initiated later in the same nest or reduce the fitness of the fledglings (Rendell & Verbeek, 1996). In some species, nest reuse can also result in later egg laying and also smaller clutches, which automatically leads also to lower reproduction success (Otterbeck et al., 2019). However, reusing nests can also have advantages. For example, several studies agreed that nest reuse can reduce the time and effort required for nest site selection, and that eggs can be laid earlier than in newly built nests (e.g. Batisteli et al., 2021; Cavitt et al., 1999; Mazgajski, 2007). Despite the higher risks, nest reuse within one season is a common phenomenon in cavity‐nesting passerines such as tits and sparrows (Tomás et al., 2007; Wesołowski, 2006), likely due to limited nesting possibilities (Wiebe, 2011). Similarly, in some raptor species nest reuse in subsequent years is frequent; often explained by lack of nesting sites and by saving time and energy (Jiménez‐Franco et al., 2014). In contrast, nest reuse is rare in open‐cup nesting passerines. So, far most cases of nest reuse were reported in Sylviid warblers (e.g., Mérő & Žuljević, 2019; Tomkins et al., 2015; Zieliński, 2012), and very few cases were also found in finches (e.g., Hafstad et al., 2005). Open‐cup nesting passerines are more vulnerable to predation than cavity nesters (Martin, 1995), and nest reuse can be highly risky mainly due to predators such as crows who are able to memorize nest position (Ibáñez‐Álamo et al., 2015; Mainwaring et al., 2015; Weidinger, 2010). In open‐cup nesting passerines, individuals with successful breeding outcomes often display nest‐site fidelity (Hoover, 2003), whereas nesting failure can result in a change of nesting site (Haas, 1998). This pattern is consistent with the “win‐stay, lose‐switch” concept (Batisteli et al., 2021; Chalfoun & Martin, 2010). The “win‐stay‐lose‐switch” strategy can be influenced by environmental changes (Kloskowski, 2021), or it can vary between sexes, e.g., while females display site‐fidelity after successful breeding, males display territory‐fidelity, or it can happen that only one sex shows site fidelity after successful breeding (Sedgwick, 2004). However, there are species in which the “win‐stay, lose‐switch” concept is not utilized, and they tend to use nesting sites, or nests repeatedly after nesting failure, i.e., following the “always stay” concept (Switzer, 1993). In some species, individuals can show consistent and inconsistent behavior with the “win‐stay, lose‐switch” strategy, i.e., some pairs follow the strategy, while some pairs do not (Kokko et al., 2004).

The Great Reed Warbler *Acrocephalus arundinaceus* inhabits reed habitats with water in Europe and the Western Palearctic, from Turkey in the south to the Scandinavian peninsula in the north, from France in the west to Mongolia in the east (Cramp, 1998). This species prefers canals, ponds, fish ponds, or shallow lakes with intermediate water levels with reed vegetation, rich in reed edges adjacent to water (Mérő et al., 2014, 2018). Offspring survival is strongly influenced by nest predation, which can occur especially during adverse weather circumstances (Mérő et al., 2014). The nesting success of Great Reed Warbler can also be negatively influenced by brood parasitism by Common Cuckoos *Cuculus canorus* (e.g., host egg ejection by adult Cuckoo, or egg or nestling eviction by Cuckoo young), especially in reed habitats with many close perches for brood parasites such as trees, shrubs and electric wires, where 50%–60% of the nests suffer from brood parasitism (Zölei et al., 2015). Similarly to other open‐cup nesting passerines (e.g., Eurasian Blackcap *Sylvia atricapilla*, Zieliński, 2012; Dunnock *Prunella modularis*, Tomkins et al., 2015), Great Reed Warblers are known to initiate new clutches in newly built nests after predation, clutch damage, or desertion of their first nest (e.g., Mérő et al., 2014). A handful of previous studies have reported on unusual nesting cases in the Great Reed Warbler, such as new nests constructed below old nests, nest reuse after brood parasitism, and quintuple brood parasitism of a nest (Hafstad et al., 2005; Marton, 2021; Mérő & Žuljević, 2019). In this study, we report on three cases of nest reuse in the Great Reed Warbler and provide potential explanations for their occurrence.

**FIGURE 1:**
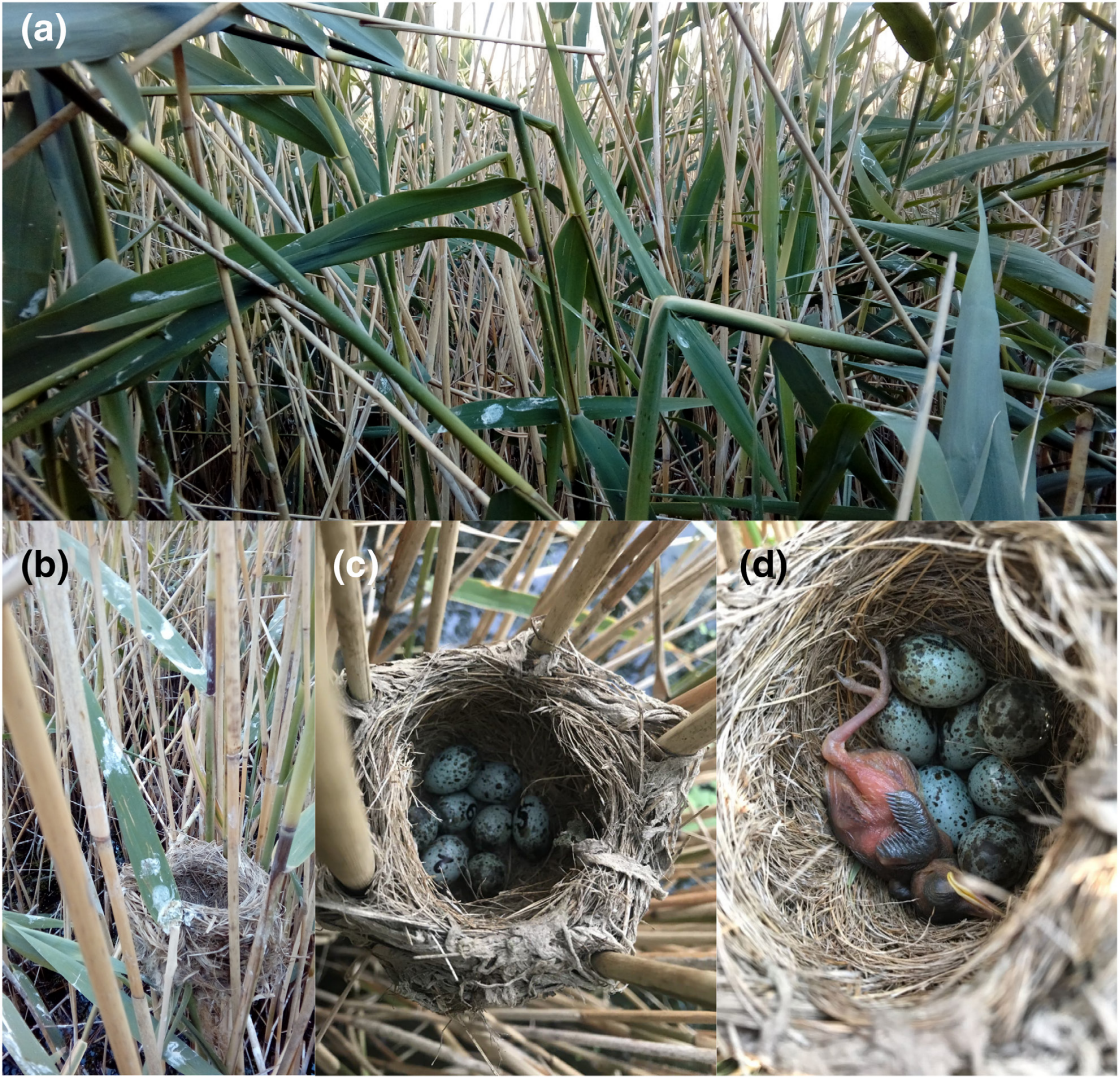
Reed damage (a), and nest desertion (b) due to en mass roosting of Starlings (mining pond near Gakovo in 2019). Great Reed Warbler nest with initial and replacement clutch in the same nest (nest reuse) with eight eggs (c), and later with a hatched five‐day‐old nestling and seven unhatched eggs (d) at the Veliki Bački Canal at the Fernbach farm.

Our study area is located in the region of Sombor, in the north‐west Serbia, in an intensive agricultural area with a moderate continental climate. In our field studies, we monitor Great Reed Warblers on mining ponds, marshes, and different types and sizes of canals (Mérő et al., 2020). Here, we report on observations on three extreme cases of nest reuse in the Great Reed Warbler; two observed on a mining pond (hereafter MP; N 45.8988°, E 19.0798°) near the village of Gakovo, and one observed on the large canal Veliki Bački Canal, at the former Fernbach farm near the town of Sombor (hereafter VBC; N 45.7332°, E 19.1798°). In MP, 40% of the area of the reed was burned in early spring by the locals. During the first half of June, we recorded en mass roosting of young Starlings *Sturnus vulgaris* (up to c. 1000 individuals), in the reed patches with presence of water (Figure 1a,b). Methods of nest surveys, nest checks and measurement of water depth taken during every visit, nest height and reed density is described in detail in Mérő, Žuljević, Varga, and Lengyel (2016); Mérő et al. (2020). Eggs were numbered with a permanent felt pen immediately after finding them in the nest during every nest checks. When we found evidence that a nest failed, we still checked it on one or two later visits, up to 10–12 days afterwards, to make sure of its fate. The rate of nest failure (due to brood parasitism, predation, and desertion) was calculated as the ratio of the failed nests divided by the total number of nests at the site. Nesting success was calculated based on the improved Mayfield (1975) method (Johnson & Shaffer, 1990).

## 2 RESULTS

We found and monitored a total of 1607 nests of Great Reed Warblers between 2008 and 2021. In MP, the mean brood parasitism rate was 4%, and predation rate was 25%. Desertion, caused mainly by the en mass roosting of Starlings, occurred in 22% of the nests. In VBC, the nests suffered from a mean parasitism rate of 42%, mean desertion rate of 24%, and mean predation rate of 16%. Mayfield nesting success was 0.25 in MP in 2019, and 0.27 in the studied section of VBC in 2021.

Re‐nesting case 1: The nest (ID: 6/2019) was found on May 18, 2019 in MP when nest construction was finished. The nest had no eggs, was built c. 30 cm from the edge of dense reed (398 stems per m^2^), with a mean water depth of 36 ± SD 4.1 cm. On May 23, we found four eggs, and on 28 May, the clutch was complete with five eggs, and the female was observed in incubation. On June 8, we found an empty nest in good condition, which suggested that the eggs were depredated. On June 12, we found three newly laid eggs, which were again found depredated (in an empty nest) on June17. On June22, the nest was still empty. On June 27, the nest contained five freshly laid eggs, which provided evidence that the nest has been reused twice. On July 9, there were four nestlings and one unhatched egg in the nest. The four‐ringed nestlings fledged successfully on July 18. The female and male were marked with color rings, which we regularly identified later during nest checks.

Re‐nesting case 2: We found evidence of a one‐time reuse of a nest (ID 13/2019, reed density 358 stems per m^2^, c. 50 cm from reed edge, mean water depth 52 ± 4.3 cm), similar to the cases described in Mérő and Žuljević (2019). This nest was found without eggs on June 12. On June 17, the nest contained four eggs, which were depredated by June 22. On June 27, we recorded four new, freshly laid eggs, which were depredated by July 12. The female and male were marked with color rings; during nest visits we recorded the same parents when they came to defend the nest.

Re‐nesting case 3: The nest (ID: 6/2021) was found with two eggs c. 60 cm from the reed edge on May 27, 2021 in VBC, where reed density was 358 stems per m^2^, and mean water depth was 31 ± 6.9 cm. On June 2, we recorded five eggs (hereafter old eggs), and observed the incubating female. Although the expected date of hatching was June 12, the nest contained five eggs until June 26, when we recorded a freshly laid sixth egg. During an earlier visit on June 22, the eggs were cold. On July 4, we found two additional fresh eggs. The nest thus contained three new eggs and five old eggs (Figure 1c). On July 16, we found one nestling and seven unhatched eggs, which were later depredated (Figure 1d). In this case, we did not mark the parents, therefore, their identity remains unknown.

## 3 DISCUSSION

Such cases of nest reuses are rarely documented in open‐cup nesting passerines, and its occurrence is difficult to explain, therefore, we carefully provide possible suggestions on why such cases of nest reuse may occur. Extreme circumstances such as intense predation pressure, early spring reed burn, and en mass roosting of young Starlings in MP may have forced the females to implement a new strategy to increase the survival chances of their offspring. In general, water level was below average in 2019 in MP, which increased the possibility of predation because nests were more accessible to mammalian predators. Water disappeared or became very shallow below many nests, which provided excellent feeding possibilities for mammals that usually avoid water (Red Fox *Vulpes vulpes* and Least Weasel *Mustela nivalis*; Mérő et al. in preparation). Reed burning considerably reduced nesting opportunities because the fire reached the reed edges adjacent to water. The lack of safe‐nesting sites can force birds into nest reuse as has been reported in Blackbirds *Turdus merula* (Wysocki, 2004). The roosting flocks of starlings damaged the reed patches by breaking the reed stems, and producing large amounts of droppings that covered reed stalks and nests, thus resulting in unsuitable nesting conditions for the Great Reed Warblers (Mérő, Žuljević, & Lengyel, 2016), which effectively ruled out the possibility of raising offspring and re‐nesting in case of nesting failure (Figure 1a,b). Under such circumstances and high density of breeding pairs, some pairs may be forced to reuse their old nests (Cancellieri & Murphy, 2013; Herzog et al., 2018). However, the year 2019 was not exceptional with regard to the density of breeding pairs compared to other years. It thus seems that nest reuse occurred to reduce the time required for nest site selection and nest construction (Cavitt et al., 1999; Redmond et al., 2007).

Laying fresh eggs into an old existing clutch at VBC was also an extreme case of nest reuse, and to our knowledge, this phenomenon has been rarely reported in the literature for open‐cup nesting passerines. In Romania, Hafstad et al. (2005) found a similar case of nest reuse in the Great Reed Warbler, when they experimentally tested host adaptations against parasitism by the Common Cuckoo by adding eggs of Chinese Quail *Coturnix chinensis* to the clutch. After the host ejected the Quail egg and two of its own (out of five eggs), it laid two new eggs into the nest containing three old eggs. Despite the delayed laying of new eggs, we believe that this behavior differs from our case. In our case, the nest contained only Great Reed Warbler eggs, and we did not document any interference with brood parasitic Cuckoos, while in Hafstad et al. (2005) the female may have been influenced by the experimental manipulation. However, the nest in Hafstad et al. (2005) was not parasitized by the Cuckoo. The case in Hafstad et al. (2005) may be better explained as clutch completion after egg rejection by the host. To some extent, our case is similar to the classic clutch overlap that was observed in several bird species (e.g., pigeons, coots, tits; Hill, 1986; Hetmanski & Wolk, 2005; Surmacki & Podkowa, 2022). However, our case differs from the earlier reported cases. While clutch overlap suggests that the first clutch is still active, eggs are incubated or nestlings are fed by the parents, while they lay a new clutch (Burley, 1980; Hays, 1984; Logan et al., 1990), in our case, the first clutch was inactive. The first egg of the later clutch was laid 14 days after the original hatching date of the first clutch. In our case, we assume that eggs (old and two new) may have been infertile, e.g. due to poor male quality (Lifjeld et al., 2007) or inexperience of a young female (Daniels & Walters, 2000), in maintaining the clutch during adverse weather conditions. The old eggs may have suffered damage because temperatures were far below average in the end of May and the beginning of June 2021, and the new eggs could also suffer damage from occasional heavy rainfalls in the first half of July. There is also a possibility that the female was absent from the nest for longer time periods during the incubation for some reason (e.g., presence of predators). Furthermore, incubation of eight eggs in the nest is probably less effective in a passerine which lays 3–5 eggs on average (e.g., Bensch, 1996; Mérő et al., 2014, 2015), as the incubation of larger clutches requires more energy for maintaining at incubation temperature (Liu et al., 2018; Reid et al., 2002). Thus, we suggest that it is possible that two of the new eggs did not receive constant heat during incubation, and therefore, may have failed to produce nestlings (Potti & Merino, 1996).

The reuse of the nest and the laying of new eggs among the old eggs may be similarly explained as in the case of MP, i.e., to save time and energy. In addition, we believe that in this case the high pressure of brood parasitism by Cuckoos in the VBC (parasitism rate c. 40%, Mérő et al., in revision), also played a role. In highly parasitized populations, Great Reed Warblers tend to develop strategies such as egg recognition and ejection to defend against brood parasitism (Moskát et al., 2008). Laying new eggs in an earlier completed clutch or in a nest that failed earlier might enable host birds to deter parasitism as previously used nests containing complete clutch may be unattractive to Cuckoos. Normally, Cuckoos parasitize host nests with incomplete clutch, usually containing three or fewer host eggs, to ensure earlier hatching of their young than the chicks of host so that the Cuckoo young can easily evict its foster siblings (Geltsch et al., 2016; Wang et al., 2020). Recognizing the advantages of nest reuse suggests adaptive flexibility in the individual and may also suggest the vigilance of individuals in the parasitized population. For example, nest reuse in the Pale‐breasted Thrush *Turdus leucomelas* reduced the chances of parasitism by Shiny Cowbirds *Molothrus bonariensis*, suggesting that thrushes followed the “win‐stay, lose‐switch strategy” (Batisteli et al., 2021).

It is common in the three reported cases that nests were located in intermediate water depth and dense reed. Nests concealed among dense reed and above water provide safer nesting circumstances (Mérő & Žuljević, 2014), because predators tend to have difficulties to find such nests and the nest defense behavior of the Great Reed Warbler was observed to be more intensive in dense reed (Mérő & Žuljević, 2017), than in sparse reeds. Water availability in reed‐beds decreases the risks of predation (Mérő et al., 2020), while nests in dense reed are more difficult to detect by the brood parasites (Mérő & Žuljević, 2017). Nest reuse in other species may occur more often in nests that are well concealed, indicating that concealment is a key factor when a nesting site is selected (Wysocki, 2004). All the three cases reported in this study suffered from nest predation. In case 1, the first clutch and the clutch from first nest reuse were depredated, in case 2, the first clutch and the clutch from first nest reuse were depredated, and in case 3, the first clutch as well as the clutch from the nest reuse were depredated in the VBC. The three reported cases are inconsistent with the predator avoidance hypothesis, and as well with the “win‐stay, lose‐switch” concept, suggesting a risky decision by the parents. Animals that experienced predation usually tend to modify and adapt their behavior to minimize the risk of losing their reproductive effort to predators (Grobis et al., 2013; Sommers & Chesson, 2019).

Adaptive behavior is crucial in the evolutionary processes in animals (Valdovinos et al., 2010). Individuals that are frequently faced with brood parasitism and predation may develop strategies to decrease the possibility of offspring loss. For example, some bird species learned that incorporating snake skins into their nests can decrease the risks of predation (Liu & Liang, 2021). In addition, egg recognition and rejection by the host is one of the most efficient tactics in deterring Common Cuckoo brood parasitism (e.g., Honza & Moskát, 2008). Analogously, the nest reuse in passerines may minimize the attention of the brood parasite, by making used nests unattractive. However, we note that nest reuse as an adaptive behavior is rare in open‐cup nesting passerines and that we recorded only three cases out of 1607 nests. This is presumably because the abundant availability of nesting sites and nesting material (Cancellieri & Murphy, 2013). Nest building behavior stimulates ovulation in the open‐cup nesting passerines, and this mechanism may repress nest reuse behavior (Boves et al., 2013; Cheng & Balthazart, 1982). Another reason for not using old nests might be if parents avoid laying new eggs into nests depredated earlier (Styrsky, 2005). Finally, the avoidance of nest reuse reduces the probability that eggs and nestlings are attacked by nest parasites that had accumulated during the earlier nesting attempts (Rendell & Verbeek, 1996).

The nest reuse in the Great Reed Warbler in this study and in Mérő and Žuljević (2019) did not provide clear evidence to support the predator avoidance hypothesis as eventually all three nests were depredated, these cases are thus more likely to support the time/energy saving theory (Cancellieri & Murphy, 2013), and anti‐parasitic behavior. We encourage scientists to collect and publish rare cases of nest reuses in passerines, to explore why these phenomena appear, and to gain more knowledge on the background of their nature.

## AUTHOR CONTRIBUTIONS

**Thomas Oliver Mérő:** Conceptualization (lead); investigation (lead); writing – original draft (equal); writing – review and editing (equal). **Antun Zuljevic:** Investigation (supporting); writing – review and editing (equal). **Olga Kolykhanova:** Investigation (supporting); writing – review and editing (equal). **Szabolcs Lengyel:** Conceptualization (supporting); writing – original draft (equal); writing – review and editing (equal).

## CONFLICT OF INTEREST

The authors claim no conflict of interests.

## Data Availability

Data sharing not applicable to this article as no datasets were generated or analysed during the current study.
